# ANXA2^+^ Small Extracellular Vesicles Drive Chemoresistance in Anaplastic Thyroid Cancer by Promoting XRCC5 Lactylation and Enhancing Non‐Homologous End‐Joining Repair

**DOI:** 10.1002/advs.76402

**Published:** 2026-07-03

**Authors:** Shanshan Su, YiShan Xiong, Yuxuan Liang, Xiang Min, Daofeng Dai

**Affiliations:** ^1^ Jiangxi Otorhinolaryngology‐Head and Neck Surgery Institute, Department of Otorhinolaryngology‐Head and Neck Surgery, The First Affiliated Hospital, Jiangxi Medical College Nanchang University Nanchang Jiangxi China

**Keywords:** anaplastic thyroid carcinoma, lactylation, non‐homologous end‐joining repair, small extracellular vesicle, XRCC5

## Abstract

Anaplastic thyroid carcinoma (ATC) is an exceptionally aggressive malignancy with dismal survival, largely due to intrinsic cisplatin resistance. This study identifies a novel mechanism by which small extracellular vesicles (sEVs) promote chemoresistance by enhancing DNA repair via protein lactylation. ATC cells secrete sEVs enriched with Annexin A2 (ANXA2). Upon delivery to recipient ATC cells, ANXA2 stabilizes the interaction between SRC kinase and lactate dehydrogenase A (LDHA), leading to increased LDHA phosphorylation (Y10), enzyme activity, and lactate production. The resulting lactate surge serves as a substrate for lysine lactylation. Ku80 (XRCC5) is identified as a key lactylation target at K265, catalyzed by the acyltransferase KAT5. This lactylation modification strengthens the interaction between Ku80 and its partner Ku70 (XRCC6), stabilizing the initial DNA‐end binding complex in the non‐homologous end‐joining (NHEJ) repair pathway. Consequently, NHEJ efficiency is significantly enhanced, enabling ATC cells to rapidly repair cisplatin‐induced DNA double‐strand breaks and survive treatment. Genetic disruption of the XRCC5‐K265 lactylation site or pharmacological inhibition of LDHA sensitizes ATC xenograft tumors to cisplatin, while in vitro, inhibition of the SRC/LDHA axis produces a similar chemosensitizing effect. This work unveils the ANXA2^+^ sEV/SRC/LDHA/lactate/XRCC5‐lactylation axis as a critical driver of NHEJ‐mediated chemoresistance in ATC, offering new potential therapeutic targets.

## Graphical abstract

ANXA2^+^ sEVs promote cisplatin resistance in ATC by stabilizing the SRC/LDHA interaction, increasing LDHA phosphorylation and activity, and lactate production. Elevated lactate levels promote KAT5‐mediated lactylation of XRCC5 at lysine 265, enhancing its binding to XRCC6. This strengthens the NHEJ DNA repair pathway, allowing rapid repair of therapy‐induced DNA damage and cell survival.

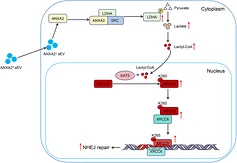

## 1 Introduction

Thyroid carcinoma constitutes the most prevalent form of endocrine cancer, with a gradually increasing incidence observed globally [1]. Anaplastic thyroid carcinoma (ATC), accounting for roughly 2% of thyroid cancers, is among the most aggressive human malignancies [2]. It is responsible for over half of all thyroid cancer deaths and carries a median survival of approximately six months from diagnosis. Cisplatin is commonly used in ATC treatment, yet their efficacy remains limited. This ineffectiveness strongly suggests the involvement of one or more underlying cellular mechanisms of chemotherapy resistance, which are not yet fully understood [3].

Small extracellular vesicles (sEVs), ranging from 30 to 150 nm in diameter, are a subtype of extracellular vesicles (EVs) that facilitate intercellular communication by transferring molecular cargo—including nucleic acids, proteins, and lipids—from donor to recipient cells. This cargo delivery enables sEVs to significantly influence key tumorigenic processes [4, 5]. Cancer stem cell‐like cells‐derived exosomal CDKN2B‐AS1 stabilizes CDKN2B to promote the growth and metastasis of thyroid cancer via TGF‐β1/Smad2/3 signaling [6]. Annexin A6 was transferred from gemcitabine‐resistant to chemotherapy‐sensitive breast cancer cells via sEVs, where it induced chemoresistance by inhibiting EGFR ubiquitination and degradation [7]. Cancer stem cell‐derived sEVs promote chemoresistance in non‐small cell lung cancer by selectively sorting pY105‐PKM2, a process mediated by the adaptor protein IQGAP1 [8].

The Warburg effect is a significant driver of tumorigenesis. It refers to the phenomenon in which tumor cells preferentially metabolize glucose via the glycolytic pathway rather than through mitochondrial oxidative phosphorylation to generate energy, even under aerobic conditions [9]. This metabolic shift leads to substantial lactate accumulation, where lactate is not merely a metabolic byproduct but also a crucial metabolic intermediate and signaling molecule. Key enzymes in the glycolytic pathway—lactate dehydrogenase (LDH)—play pivotal roles in regulating lactate levels [10]. As a novel form of post‐translational modification, lactylation directly utilizes lactate as its substrate. Thus, lactate levels directly influence the extent of lactylation. This modification is catalyzed by “Writer” enzymes, which add lactyl groups. Known lactylation “Writer” proteins primarily include histone acetyltransferases such as P300 [11], GCN5 [12], TIP60 [13], KAT8 [14], AARS1 [15], and AARS2 [16]. Lactylation modification occurs on both histones and non‐histone proteins, with the functional mechanisms of non‐histone lactylation being more diverse. ALDH1A3 promotes the tetramerization of PKM2, enhancing its pyruvate kinase activity, resulting in increased lactate levels and elevated lactylation at the K247 residue of XRCC1 in glioblastoma stem cells. This facilitates XRCC1 nuclear translocation, enhances DNA repair, and confers therapeutic resistance in glioblastoma [17]. How lactylation modification influences the chemoresistance in ATC has not yet been reported.

Non‐homologous end joining (NHEJ) is the primary pathway for repairing double‐strand breaks (DSBs) throughout the cell cycle, accounting for nearly all DSB repair outside of the S and G2 phases [18]. NHEJ relies on the Ku complex (Ku70/Ku80) binding to the damaged DNA ends, thereby enhancing the affinity of the NHEJ enzymatic components, which include polymerases (Pol µ and Pol λ), nucleases (the Artemis/DNA‐PKcs complex), and ligases (the XLF/XRCC4/Lig4 complex). DSBs are first recognized by the Ku complex [19]. The Ku complex acts as a loading factor, recruiting other NHEJ proteins to facilitate the joining of DNA ends. DNA‐PKcs has a high affinity for DNA ends, and this affinity is further enhanced when the Ku complex is bound to the ends [18]. The nuclease Artemis tightly associates with DNA‐PKcs and is likely recruited to DSBs together with DNA‐PKcs to perform its nuclease function. Nucleotide addition is carried out by the polymerases Pol µ and Pol λ. Finally, the XRCC4/Lig4 complex is responsible for the critical DNA ligation step.

Here, we report that protein lactylation regulates the function of XRCC5 (Ku80) during NHEJ by promoting its interaction with XRCC6 (Ku70). ANXA2^+^ sEVs deliver ANXA2 into recipient ATC cells, where it stabilizes the interaction between SRC and LDHA to elevate the phosphorylation of LDHA. The subsequent increase in lactate production promotes XRCC5 lactylation, which strengthens the XRCC5/XRCC6 interaction and boosts NHEJ repair efficiency, ultimately leading to chemoresistance in ATC cells. Furthermore, inhibiting XRCC5 lactylation increases the sensitivity of ATC cells to chemotherapy.

## 2 Results

### 2.1 Lactylation Promotes NHEJ Repair and Chemoresistance

The role of lactylation in ATC is not yet defined, despite its established importance in chemoresistance for several other cancers. As shown in Figure 1A, ATC exhibited significantly higher pan‐lactylation levels than benign thyroid nodules (BTN). As the major enzyme responsible for lactate production [20], LDHA may thus promote protein lactylation via increasing its substrate availability [11]. Therefore, we also assessed LDHA expression via IHC in BTN and ATC tissues, and observed a similar pattern (Figure 1B). Both lactate accumulation and pan‐lactylation levels showed a graded elevation from normal thyroid epithelial cells (Nthy‐ori 3‐1) to PTC (TPC‐1, BCPAP) and further to ATC cells (8305C, CAL‐62) (Figure S1A,B). Treatment with the LDHA inhibitor oxamate reduced both lactate and pan‐lactylation levels in 8305C and CAL‐62 cells (Figure 1C,D). In contrast, adding sodium lactate (NALA) counteracted this effect and rescued pan‐lactylation levels from the reductions (Figure 1D). Treatment with cisplatin increased pan‐lactylation levels in 8305C and CAL‐62 cells (Figure S2). Comet assay results indicated that oxamate treatment significantly increased the tail moments of 8305C and CAL‐62 cells following 8 h of cisplatin treatment, and this elevation was eliminated by further NALA treatment; little difference was observed after 0.5 h of cisplatin exposure (Figure 1E). Treatment with oxamate significantly reduced cellular NHEJ levels, and this reduction was reversed by the further addition of NALA (Figure 1F). This inhibitory effect of oxamate, which was also rescued by NALA, was corroborated by an independent cell‐free biochemical NHEJ assay​ using plasmid DNA as the substrate, where repair efficiency was quantified by gel electrophoresis (Figure 1G). The oxamate treatment rendered ATC cells sensitive to cisplatin, but further treatment with NALA reversed this effect (Figure 1H).

**FIGURE 1:**
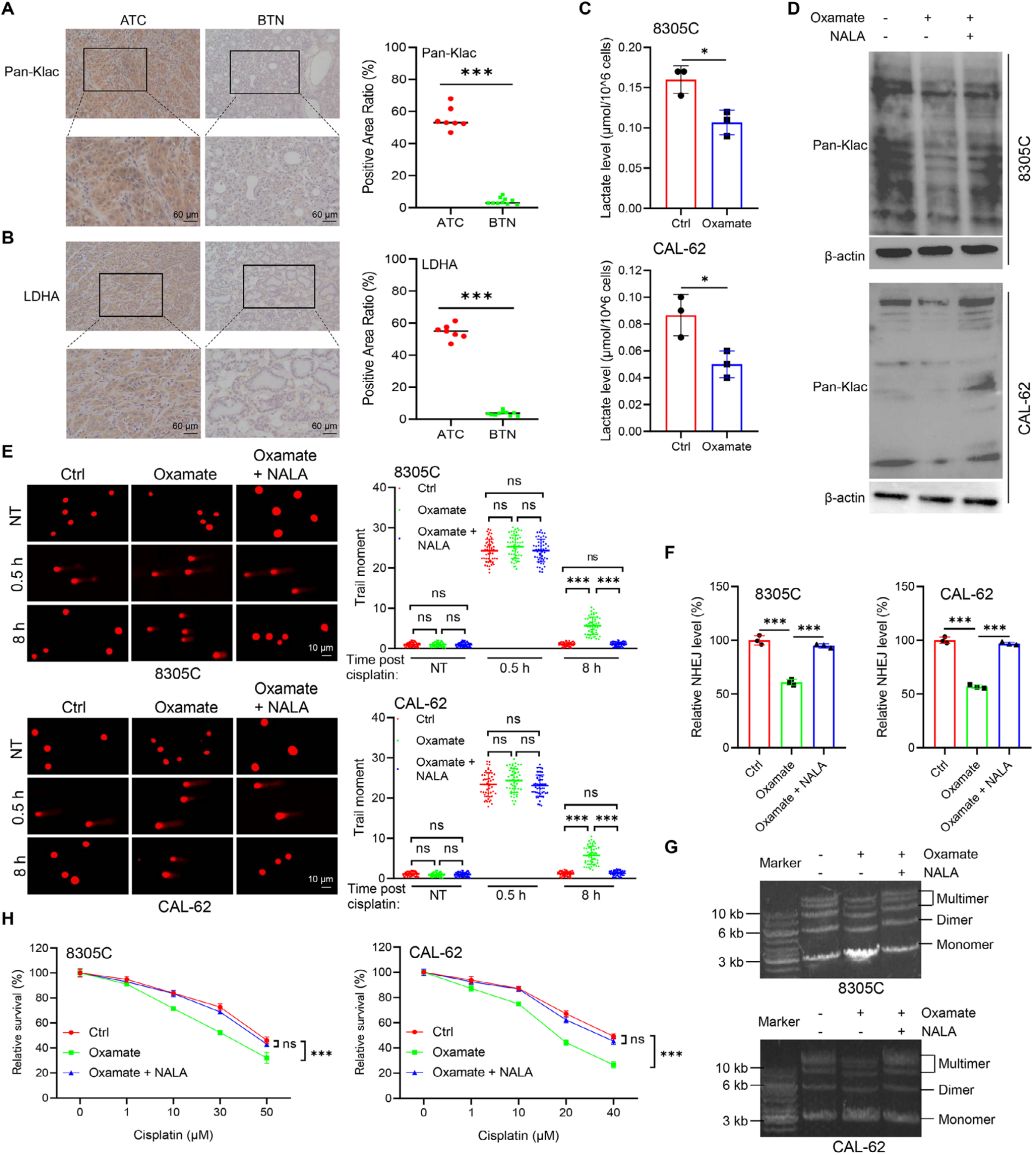
Lactylation promotes NHEJ repair and chemoresistance. IHC analysis of pan‐lactylation level (A) and LDHA expression (B) in ATC (*n* = 7) and BTN (*n* = 10) tissues. The data were analyzed using the Student's *t*‐test. (C) The lactate levels were analyzed in 8305C and CAL‐62 cells treated with control or oxamate (25 mM) for 24 h. *n* = 3 in each group. The data were analyzed using the Student's *t*‐test. (D) The pan‐lactylation level was analyzed in 8305C and CAL‐62 cells treated with control, oxamate, or oxamate plus NALA (10 mM) for 24 h. (E) ATC cells treated as above were collected for comet assays at indicated time after cisplatin (12 µM) treatment. NT, no treatment. *n* = 50 in each group. The data were analyzed using one‐way ANOVA. Scale bars, 10 µm. (F) The cellular NHEJ level in 8305C and CAL‐62 cells subjected to the above treatment. *n* = 3 in each group. The data were analyzed using one‐way ANOVA. (G) The cell‐free NHEJ activity in 8305C and CAL‐62 cells subjected to the above treatment. (H) Dose‐response curves for cisplatin in ATC cells treated with control, oxamate, or oxamate plus NALA (25 mM) for 72 h. *n* = 3. The data were analyzed using two‐way ANOVA.

### 2.2 XRCC5 is Lactylated at Residue K265 by KAT5

Screening of key NHEJ proteins, including XRCC4, XRCC5, and XRCC6, revealed that all were lactylated, with XRCC5 exhibiting the highest level of this modification (Figure S3A). Immunofluorescence analysis revealed that XRCC5 was primarily nuclear, while pan‐lactylation signals were detected in both the cytoplasm and nucleus (Figure S3B). Compared to BTN, ATC tissues exhibited a stronger co‐localization signal between XRCC5 and pan‐lactylation, indicative of a significantly higher lactylation level specifically on XRCC5 in ATC (Figure S3B). Despite the identification of XRCC5 lactylation by Li et al. and Jin et al. [21, 22], the precise mechanism governing this modification remains to be defined. Lactylation of XRCC5 was confirmed in 8305C and CAL‐62 cells by co‐immunoprecipitation followed by western blotting analysis (Figure 2A). Oxamate treatment decreased XRCC5 lactylation, while further addition of NALA restored it (Figure 2B). Mass spectrometry detected a lactylation modification at the K265 residue of XRCC5 (Figure 2C and Figure S4). We performed site‐directed mutagenesis (K265R) to validate K265 as the lactylation site on XRCC5. While a robust lactylation signal was detected in the XRCC5 wild type (WT), the XRCC5 K265R mutant showed a dramatic decrease in this modification (Figure 2D). Treatment with cisplatin clearly elevated lactylation of XRCC5 WT but not lactylation of XRCC5 K265R mutant in 8305C and CAL‐62 cells (Figure S5), suggesting a potential role for XRCC5 K265 lactylation in ATC chemoresistance. Substrate protein lactylation is catalyzed by upstream acyltransferases [23]. To determine which acyltransferase regulates XRCC5 lactylation, we overexpressed representative members of the KAT family in a screening approach informed by previous studies [24]. Overexpression of KAT2A or KAT5 robustly increased XRCC5 lactylation in both 8305C and CAL‐62 cells, compared with empty vector or other acyltransferases (Figure 2E). Using co‐immunoprecipitation assay, we observed that KAT2A and KAT5 each interacted with XRCC5 in both 8305C and CAL‐62 cells (Figure 2E). Overexpression of KAT2A or KAT5 markedly increased the lactylation of XRCC5; notably, the K265R mutation in XRCC5 significantly suppressed the enhancing effect induced by KAT5 overexpression, whereas it had no impact on the enhancement caused by KAT2A overexpression (Figure 2F). Depletion of KAT5 markedly reduced XRCC5 WT lactylation at K265 (Figure 2G). These results suggested that XRCC5 was lactylated at K265 by KAT5.

**FIGURE 2:**
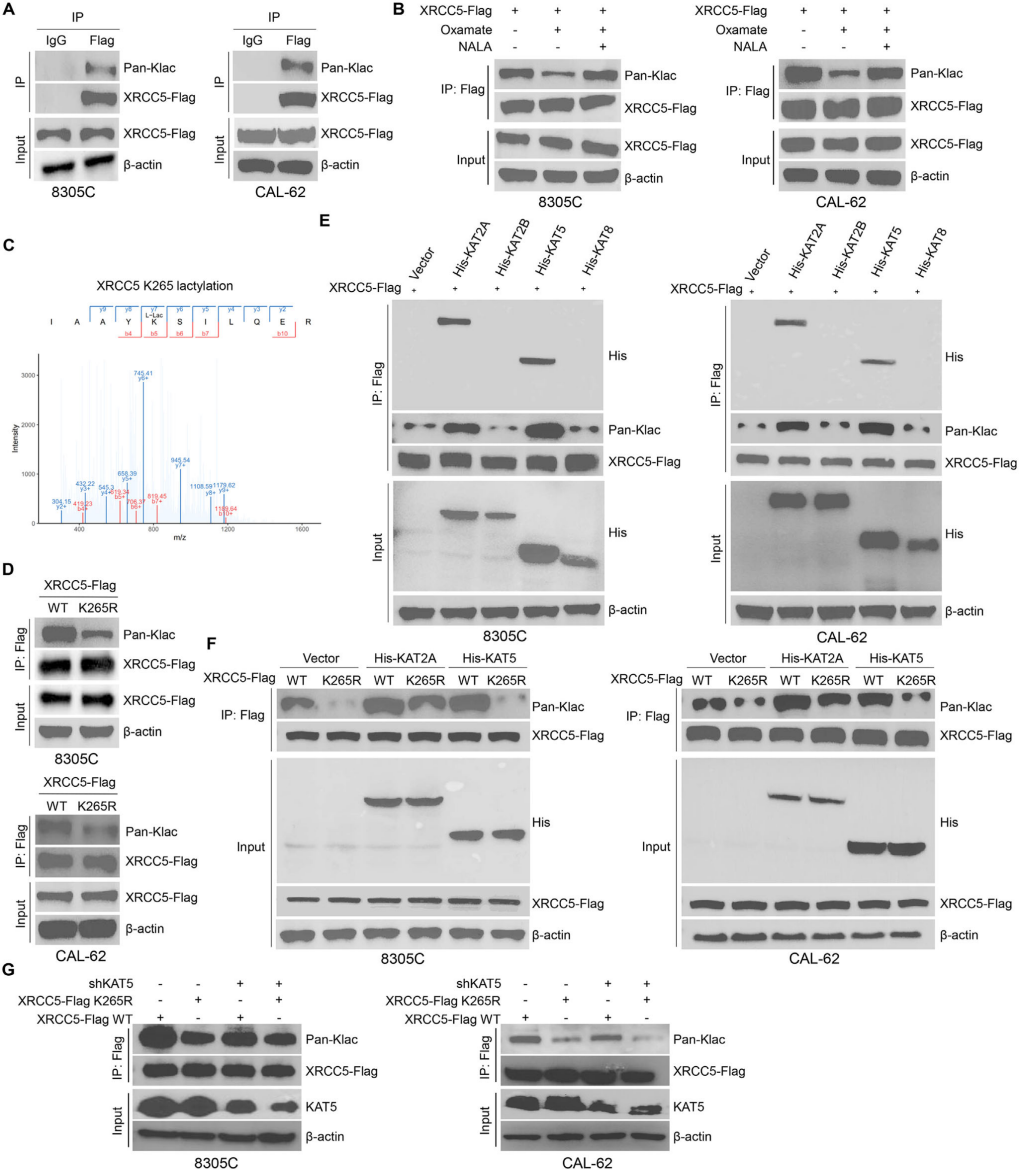
XRCC5 is lactylated at residue K265 by KAT5. (A) Cell lysates of 8305C and CAL‐62 cells were immunoprecipitated with anti‐Flag or control IgG, followed by immunoblotting with the antibody against pan‐Klac or the Flag tag. (B) Whole‐cell extracts of 8305C and CAL‐62 cells treated with control, oxamate, or oxamate plus NALA, were immunoprecipitated with anti‐Flag, followed by immunoblotting with the antibody against pan‐Klac or the Flag tag. (C) Mass spectra of the lactylated site of XRCC5 (K265). (D) Whole‐cell lysates from ATC cells expressing Flag‐tagged XRCC5 (WT or K265R mutant) were subjected to immunoprecipitation with anti‐Flag, followed by immunoblotting with the antibody against pan‐Klac or the Flag tag. (E) Lysates from 8305C and CAL‐62 cells overexpressing the indicated XRCC5‐Flag and His‐tagged acyltransferases (KAT2A, KAT2B, KAT5, or KAT8) were immunoprecipitated with anti‐Flag antibody, followed by immunoblotting with the antibody against pan‐Klac, the His tag, or the Flag tag. (F) Whole‐cell lysates from ATC cells overexpressing Flag‐tagged XRCC5 (WT or K265R mutant) and His‐tagged acyltransferases (KAT2A or KAT5) were immunoprecipitated with anti‐Flag antibody, followed by immunoblotting with the antibody against pan‐Klac or the Flag tag. (G) Lysates from ATC cells expressing Flag‐tagged XRCC5 (wild‐type or K265R mutant) under KAT5 knockdown (shKAT5) conditions were subjected to immunoprecipitation using an anti‐Flag antibody. The immunoprecipitates were then analyzed by immunoblotting with antibodies against pan‐lactylation or the Flag tag.

### 2.3 Lactylation of XRCC5 at K265 Enhances NHEJ Repair and Chemoresistance by Stabilizing the XRCC5/XRCC6 Complex

During the repair of DSBs via NHEJ, XRCC5 plays an essential role together with XRCC6 by forming the XRCC5–XRCC6 heterodimer. We first confirmed that XRCC5 indeed interacted with XRCC6 in 8305C and CAL‐62 cells using co‐immunoprecipitation (Figure 3A). Oxamate treatment attenuated the interaction between XRCC5 and XRCC6, whereas the addition of NALA counteracted this effect in ATC cells (Figure 3B). The structural analysis by Walker et al. revealed that the K265 residue of XRCC5 resided at the XRCC5–XRCC6 interface [25]. Our docking analysis based on the XRCC5–XRCC6 PDB structure indicated that K265 lactylation in XRCC5 reduced complex binding energy and elevated hydrogen bonds (Figure 3C). The interaction between XRCC5 and XRCC6 was also markedly weakened by the K265R mutation in XRCC5 (Figure 3D). Nevertheless, knockdown of KAT2A did not alter the XRCC5‐XRCC6 interaction (Figure S6), supporting the notion that KAT2A‐mediated lactylation of XRCC5 does not contribute to this specific K265‐dependent function. In NHEJ, the DSBs are first recognized by the XRCC5‐XRCC6 heterodimer [18]. To determine whether lactylation of XRCC5 at K265 regulates the recruitment of the XRCC5–XRCC6 complex to DSB sites, we assessed their chromatin enrichment after cisplatin‐induced damage. The chromatin levels of both XRCC5 and XRCC6 following cisplatin treatment were significantly lower in XRCC5 K265R mutant cells compared to XRCC5 WT cells (Figure 3E). To investigate the role of XRCC5 lactylation at lysine 265 in DNA damage repair, we generated cells in which endogenous XRCC5 was knocked down and stably expressed either XRCC5 WT or the XRCC5 K265R mutant (Figure S7). Comet assays showed that tail moments of ATC cells containing the XRCC5 K265R mutant were significantly longer than those of cells harboring XRCC5 WT after 8 h of cisplatin treatment (Figure 3F). The K265R mutation in XRCC5 resulted in a significant decrease in cellular NHEJ repair efficiency compared with XRCC5 WT (Figure 3G). This finding was confirmed by an independent cell‐free NHEJ assay (Figure S8). Overexpression of KAT5 conferred cisplatin resistance in cancer cells with XRCC5 WT but not in those with the XRCC5 K265R mutant (Figure 3H).

**FIGURE 3:**
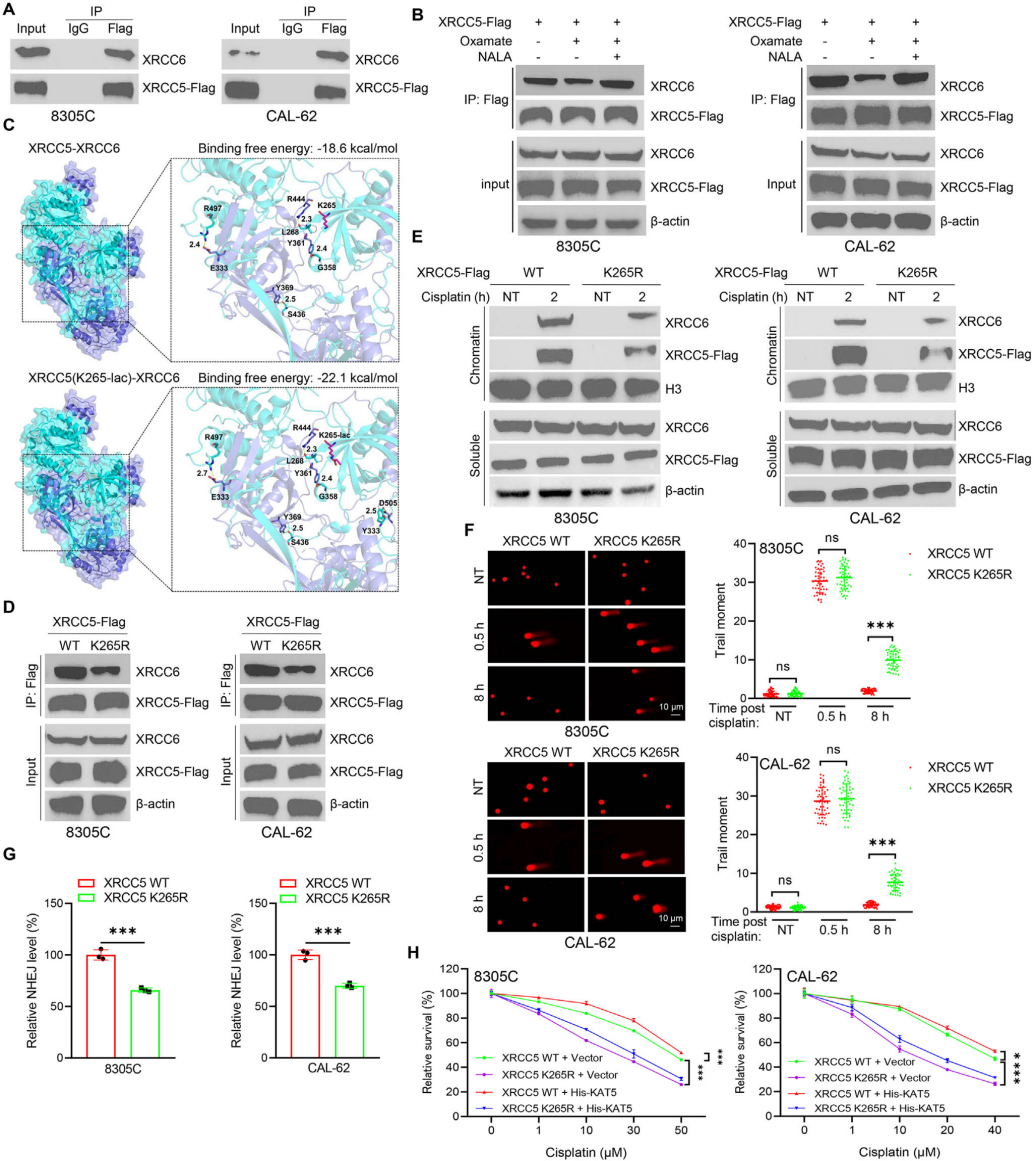
Lactylation of XRCC5 at K265 enhances NHEJ repair and chemoresistance by stabilizing the XRCC5/XRCC6 complex. (A) Cell lysates of 8305C and CAL‐62 cells were immunoprecipitated with anti‐Flag or control IgG, followed by immunoblotting with the antibody against XRCC6 or the Flag tag. (B) Whole‐cell extracts of 8305C and CAL‐62 cells treated with control, oxamate, or oxamate plus NALA, were immunoprecipitated with anti‐Flag, followed by immunoblotting with the antibody against XRCC6 or the Flag tag. (C) Molecular docking analysis of the XRCC5‐XRCC6 interaction using GRAMM, comparing the residue K265 of XRCC5 in its unmodified state versus with a lactyl modification. (D) The effect of the K265R mutation in XRCC5 on the interaction between XRCC5 and XRCC6 in ATC cells. (E) ATC cells overexpressing Flag‐tagged XRCC5 (WT or K265R mutant) were treated with cisplatin (5 µM) for 2 h. Chromatin and soluble fractions were isolated for immunoblotting with the antibody against XRCC6 or the Flag tag. (F) ATC cells in which endogenous XRCC5 was knocked down and expressed either XRCC5 WT or the XRCC5 K265R mutant were collected for comet assays at indicated time after cisplatin (12 µM) treatment. NT, no treatment. *n* = 50 in each group. The data were analyzed using the Student's *t*‐test. Scale bars, 10 µm. (G) The effect of K265 mutation in XRCC5 on NHEJ repair efficiency in ATC cell. *n* = 3 in each group. The data were analyzed using the Student's *t*‐test. (H) Survival of KAT5‐overexpressing 8305C and CAL‐62 cells expressing XRCC5‐Flag WT or the XRCC5‐Flag K265R mutant was assessed after the indicated treatments. *n* = 3. The data were analyzed using two‐way ANOVA.

### 2.4 ANXA2 Increases LDHA Enzyme Activity by Promoting the SRC/LDHA Interaction

To investigate the mechanism underlying the LDHA in ATC cells, we analyzed 8305C cells by immunoprecipitation assay using LDHA‐specific antibody (IgG antibody was used as a control group). Samples were separated by SDS‐PAGE and analyzed by LC‐MS/MS, which identified one potential interacting protein: ANXA2 (Figure 4A). IHC analysis showed that ANXA2 expression was significantly higher in ATC than in BTN tissues (Figure 4B). ANXA2 has been demonstrated to interact with SRC [26, 27]. Furthermore, SRC can phosphorylate LDHA at tyrosine residue 10 (Y10) [28, 29]. Co‐immunoprecipitation assays revealed that ANXA2, SRC, and LDHA engaged in a tripartite interaction (Figure 4C). The knockdown of ANXA2 markedly decreased the interaction between SRC and LDHA (Figure 4D). This reduction resulted in attenuated phosphorylation of LDHA at Y10, without affecting the total protein level of LDHA itself (Figure 4E). IHC analysis demonstrated increased phosphorylation of LDHA at Y10 in ATC compared to that in BTN (Figure 4F). The knockdown of ANXA2 also led to lower LDHA enzyme activity and lactate production (Figure 4G,H).

**FIGURE 4:**
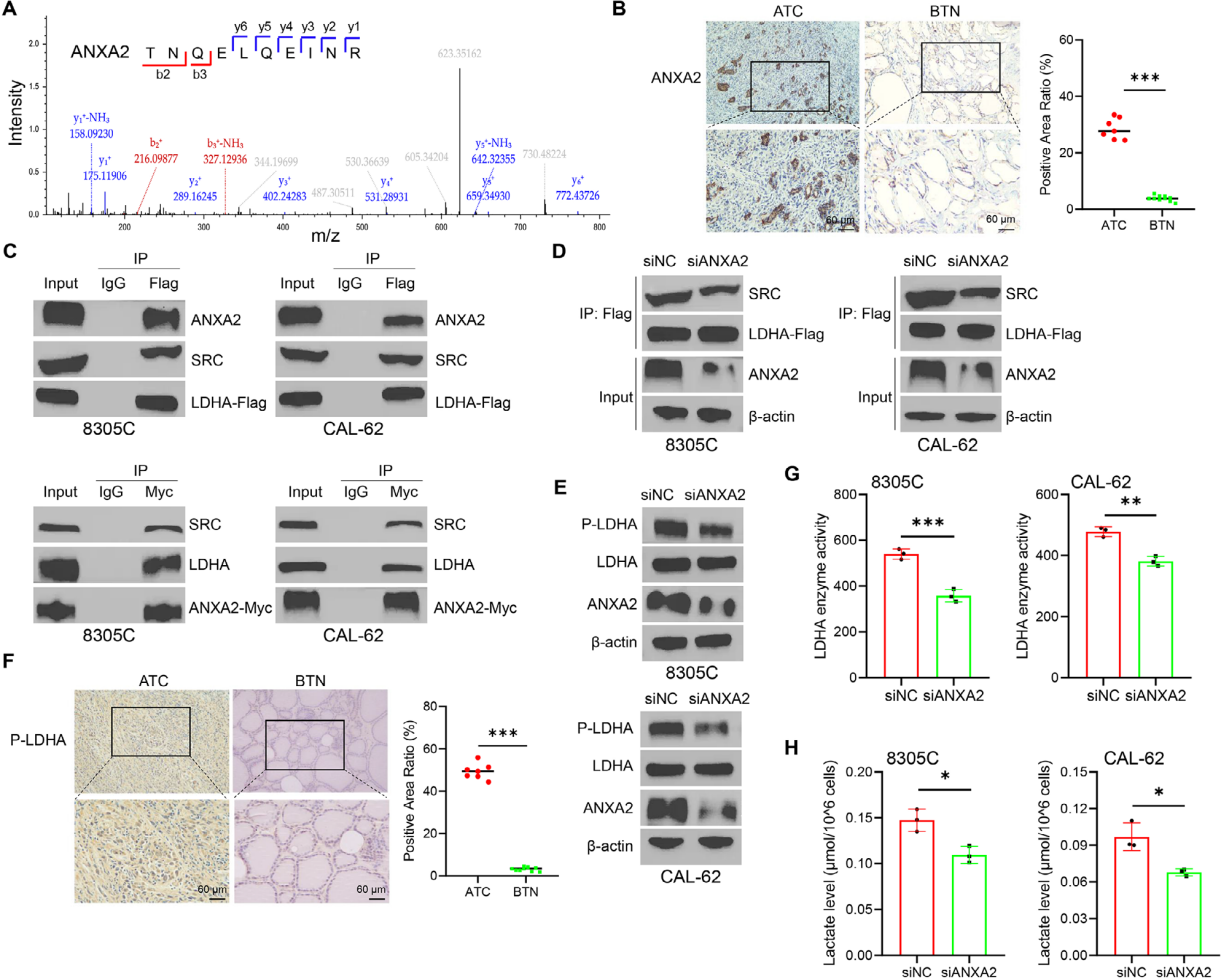
ANXA2 increases LDHA enzyme activity by promoting the SRC/LDHA interaction. (A) Mass spectra of a peptide of ANXA2 in 8305C cells. (B) IHC analysis of ANXA2 expression in ATC (*n* = 7) and BTN (*n* = 10) tissues. The data were analyzed using the Student's *t*‐test. (C) Top: Whole‐cell extracts of 8305C and CAL‐62 cells overexpressing Flag‐tagged LDHA were immunoprecipitated with anti‐Flag, followed by immunoblotting. Bottom: Cell lysates of 8305C and CAL‐62 cells overexpressing Myc‐tagged ANXA2 were immunoprecipitated with anti‐Myc, followed by immunoblotting. The effect of knocking down ANXA2 on the interaction between SRC and LDHA (D) and phosphorylation of LDHA at Y10 (E). (F) IHC analysis of LDHA phosphorylation level at Y10 in ATC (*n* = 7) and BTN (*n* = 10) tissues. The data were analyzed using the Student's *t*‐test. The effect of depleting ANXA2 on LDHA enzyme activity (G) and the lactate level (H) in 8305C and CAL‐62 cells. *n* = 3 in each group. The data were analyzed using the Student's *t*‐test.

### 2.5 ANXA2^+^ SEV Enhances NHEJ Repair and Chemoresistance by Stabilizing the SRC/LDHA Complex

Using proximity barcoding assay, we compared the sEV protein profiles of 8305C and Nthy‐ori 3‐1 cells. We found that the abundance of ANXA2 was significantly higher in sEVs derived from 8305C cells than those from Nthy‐ori 3‐1 cells​ (Figure 5A). To investigate the function of ANXA2‐enriched sEVs, the HEK293T cell was stably transduced with lentiviruses carrying either an ANXA2 overexpression construct (Lenti‐ANXA2) or a negative control (Lenti‐NC) (Figure 5B). We isolated sEVs from HEK293T cells carrying lenti‐ANXA2 or lenti‐NC, and the isolated sEVs were designated as ANXA2‐sEV and NC‐sEV, respectively. The properties of sEVs isolated from HEK293T cells were validated using TEM and western blotting. TEM confirmed the characteristic morphology of the vesicles (Figure 5C). Western blotting analysis confirmed the presence of the sEV marker TSG101 in the sEV fraction, while the negative control marker GM130 (a Golgi protein) was detected only in the whole‐cell lysate, not in the sEVs (Figure 5D). Isolated sEVs from HEK293T cells were robustly taken up by recipient 8305C and CAL‐62 cells, evidenced by the co‐localization of DiO‐labeled sEVs with DAPI‐stained nuclei of ATC cells (Figure 5E). The ANXA2‐sEV treatment notably enhanced the interaction of SRC with LDHA in ATC cells compared with the NC‐sEV treatment (Figure 5F). The treatment with ANXA2‐sEV enhanced LDHA phosphorylation at Y10, LDHA enzyme activity, and lactate production; these effects were completely abolished by the SRC inhibitor KX2‐391 (Figure 5G–I). Similarly, ANXA2‐sEV increased both the lactylation of XRCC5 and its interaction with XRCC6; these changes were also reversed by the KX2‐391 (Figure 5J,K). Furthermore, the promotion of cellular NHEJ repair efficiency induced by ANXA2‐sEV was suppressed by the SRC inhibitor (Figure 5L). These results were also confirmed by the cell‐free NHEJ assay (Figure 5M). The ANXA2‐sEV treatment rendered ATC cells resistant to cisplatin, but further treatment with the SRC inhibitor reversed this effect (Figure 5N). Treatment with cisplatin increased secretion of ANXA2^+^ sEVs in 8305C and CAL‐62 cells (Figure S9). To evaluate the effect of knocking down ANXA2 on the activity of ANXA2^+^ sEVs, we stably transduced HEK293T cells with lentiviruses carrying either an ANXA2 knockdown construct (Lenti‐shANXA2) or a negative control (Lenti‐shNC) (Figure S10A). The sEVs from HEK293T cells carrying Lenti‐shANXA2 or Lenti‐shNC were designated as shANXA2‐sEV and shNC‐sEV, respectively. The treatment with shANXA2‐sEV decreased the interaction between SRC and LDHA, LDHA phosphorylation at Y10, LDHA enzyme activity, and lactate production compared with shNC‐sEV (Figure S10B–E). The shANXA2‐sEV treatment reduced the lactylation of XRCC5 and its interaction with XRCC6, leading to impaired NHEJ repair efficiency (Figure S10F–I). Furthermore, the treatment with shANXA2‐sEV rendered ATC cells sensitive to cisplatin (Figure S10J).

**FIGURE 5:**
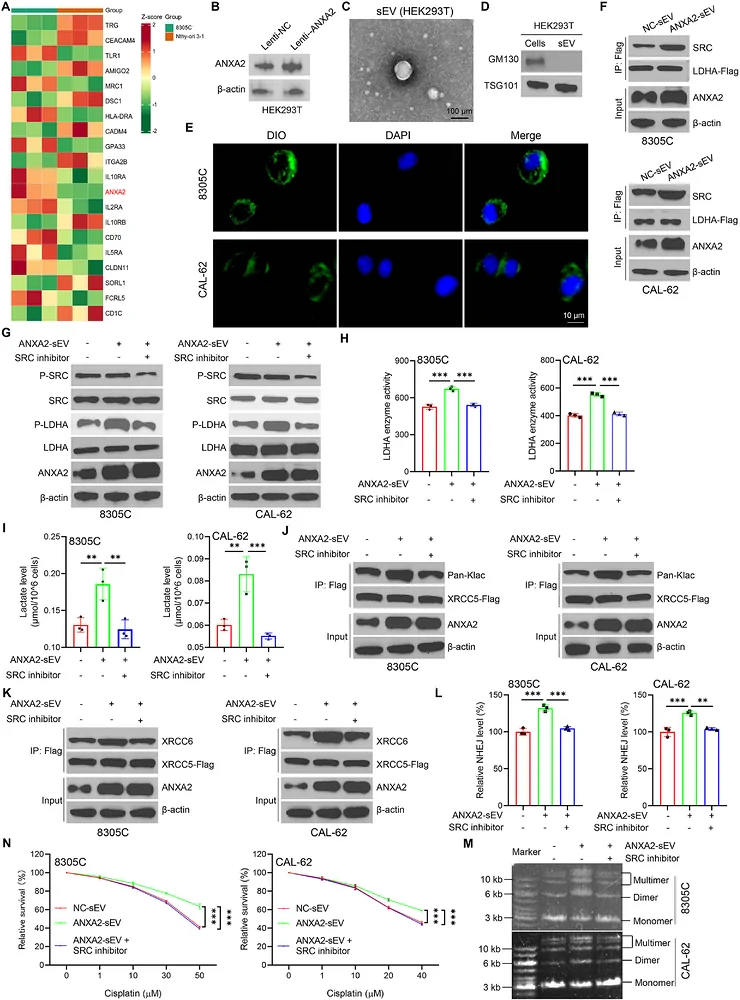
ANXA2^+^ sEV enhances NHEJ repair and chemoresistance by stabilizing the SRC/LDHA complex. (A) The heatmap displayed the top 20 differentially expressed sEV proteins between 8305C and Nthy‐ori 3‐1 cells. (B) The expression of ANXA2 in HEK293T cells stably transduced with lentiviral ANXA2 (Lenti‐ANAX2) or lentiviral NC (Lenti‐NC). NC, negative control. (C) Micrographs of sEVs isolated from HEK293T cells. (D) The expression of TSG101 and GM130 in HEK293T cells and sEVs derived from HEK293T cells. (E) Dio‐labeled sEVs from HEK293T cells were taken up by ATC cells whose nuclei were stained using DAPI. (F) The effect of ANXA2^+^ sEVs on the interaction between SRC and LDHA in ATC cells. The 8305C and CAL‐62 cells were treated with NC‐sEV, ANXA2‐sEV, or ANXA2‐sEV plus the SRC inhibitor KX2‐391 (100 nM). The effect of the above treatment on LDHA phosphorylation at Y10 (G), LDHA enzyme activity (H), lactate production (I), lactylation of XRCC5 (J), the interaction of XRCC5 with XRCC6 (K), the cellular NHEJ repair efficiency (L), the cell‐free NHEJ repair efficiency (M), and cisplatin resistance (N) in ATC cells. For panels H, I, and L, *n* = 3 per group; one‐way ANOVA was used. For panel N, *n* = 3 per condition; two‐way ANOVA was used.

### 2.6 Targeting the XRCC5 K265 Lactylation Enhances the Sensitivity of ATC Cells to Chemotherapy

To investigate the role of protein lactylation in ATC chemoresistance, we established a xenograft model by injecting 8305C cells subcutaneously into nude mice. Mice were treated with either vehicle (saline), oxamate alone, cisplatin alone, or a combination of cisplatin and oxamate. While the oxamate treatment alone did not significantly affect the growth of ATC xenografts, it markedly enhanced their sensitivity to cisplatin treatment (Figure 6A–C). Furthermore, the administration of oxamate led to a significant reduction in the proportion of Ki67‐positive cells in mice treated with cisplatin (Figure 6D). To explore the function of XRCC5 lactylation at K265 in ATC chemoresistance, we established a xenograft mouse model via subcutaneous injection of 8305C cells with endogenous XRCC5 knockdown and stable expression of either XRCC5 WT or the XRCC5 K265R mutant into nude mice. While the XRCC5 K265R mutation by itself had no significant effect on ATC xenograft growth, conversely, it rendered the tumors markedly more sensitive to cisplatin (Figure 6E–G). Moreover, the XRCC5 K265R mutation significantly abrogated the Ki67‐positive cell population in tumors from mice receiving cisplatin (Figure 6H).

**FIGURE 6:**
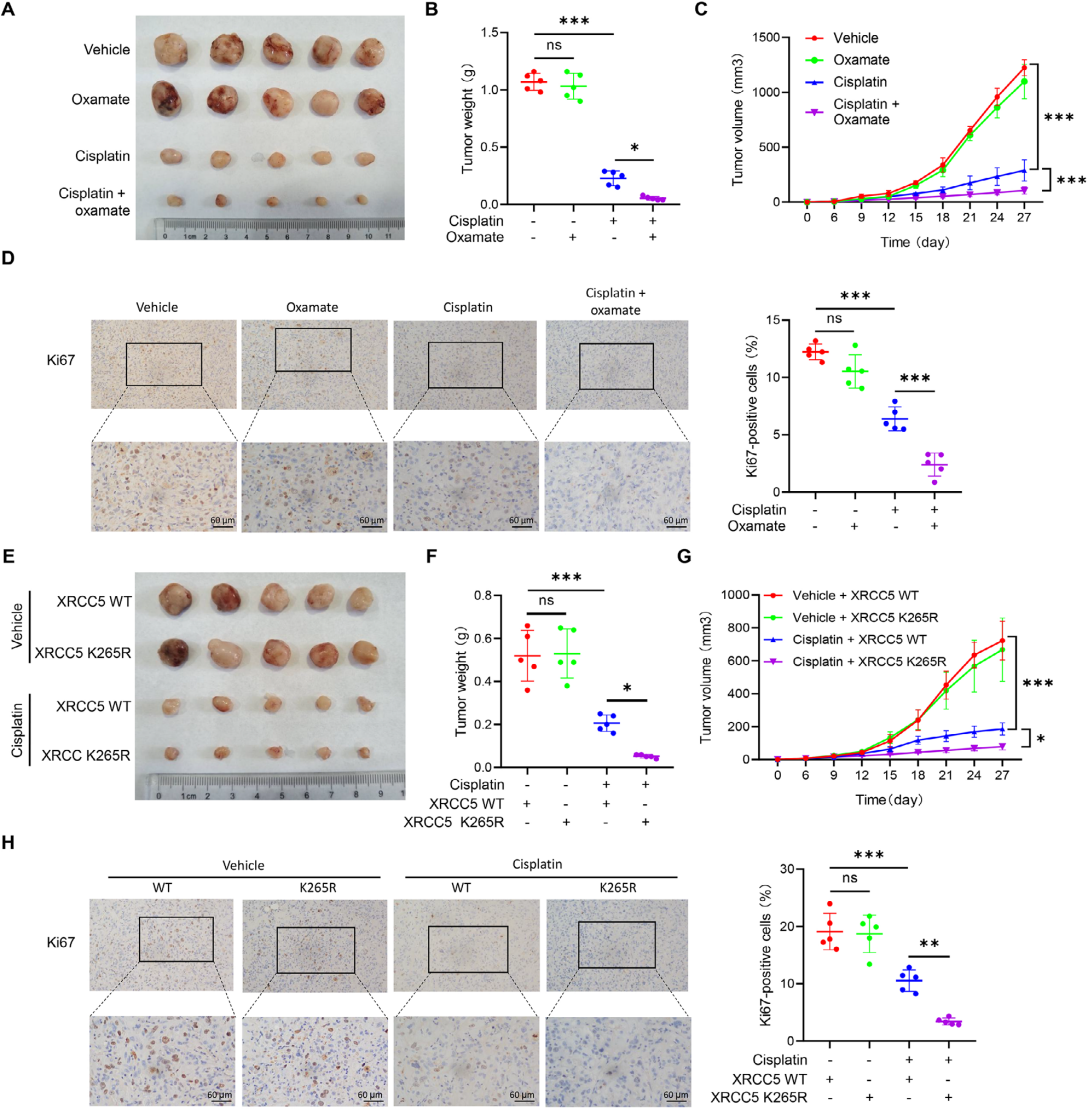
Targeting the XRCC5 K265 lactylation enhances the sensitivity of ATC cells to chemotherapy. Representative tumor images (A), tumor weight (B), and tumor volume (C) of nude mice treated with vehicle, oxamate alone, cisplatin alone, or the combination of oxamate and cisplatin. (D) The proportion of Ki67‐positive cells was analyzed in tumor tissue sections using IHC analysis. Nude mice were inoculated with 8305C cells stably expressing shRNA against endogenous XRCC5, along with reconstituted expression of either wild‐type XRCC5 (WT) or the K265R mutant. Mice were then treated with vehicle (saline) or cisplatin (2 mg/kg, once per week). (E) Representative images of tumors harvested from each group. (F) Tumor weights at the endpoint. (G) Tumor growth curves. (H) The proportion of Ki67‐positive cells in tumor sections, as analyzed by IHC. For panels B, D, F, and H, *n* = 5 mice in each group; one‐way ANOVA was used. For panels C and G, *n* = 5 mice in each group; two‐way ANOVA was used.

## 3 Discussion

The Ku heterodimer (Ku70/Ku80, XRCC6/XRCC5) forms a ring‐shaped structure that binds to DNA ends. Upon recruitment to DSBs during NHEJ, it serves to protect the DNA ends and provide a scaffold for the recruitment of the DNA‐dependent protein kinase catalytic subunit (DNA‐PKcs) [30, 31]. The eviction of the Ku heterodimer from DNA is achieved through ubiquitination of XRCC5, which targets it for removal [32, 33]. This process is regulated by specific E3 ligases. RNF138 promotes its displacement from DSB sites specifically during the S and G2 phases of the cell cycle [34], while RNF8‐dependent ubiquitination mediates the degradation of XRCC5 [34, 35]. Additionally, RNF126 has also been identified as an E3 ligase for XRCC5 [36]. The transition from crotonylation to SUMOylation at XRCC5 K568 triggers DNA‐PKcs assembly and DNA‐PKcs autophosphorylation at S2056, thereby activating DSB repair and conferring radioresistance to tumor cells [37]. SUMO2/3 promotes XRCC5 SUMOylation at K307 to confer oxaliplatin resistance in colorectal cancer [38]. However, it is unknown whether XRCC5 lactylation contributes to chemoresistance in malignant tumors, especially in aggressive cancers like ATC.

In this study, we found that XRCC5 was lactylated at K265 by KAT5 and that the lactylation of XRCC5 further hyperactivated NHEJ processes. ANXA2^+^ sEVs transferred ANXA2 to recipient ATC cells. Within recipient cells, ANXA2 stabilized SRC/LDHA interaction to promote LDHA phosphorylation, which enhanced LDHA activity, elevated lactate levels, and upregulated XRCC5 lactylation. The increased lactylation of XRCC5 enhanced its binding to XRCC6, facilitating their recruitment to DSBs, promoting NHEJ activity, and ultimately leading to chemoresistance. Unrepaired DSBs accumulated due to compromised NHEJ when LDHA was inhibited or when a K265R mutation was introduced in XRCC5. Furthermore, the XRCC5 K265R mutation increased sensitivity to cisplatin in both ATC cells and the mouse xenograft model. In summary, our findings provide proof‐of‐concept evidence that targeting XRCC5 K265 lactylation may be a promising approach to enhance chemosensitivity in ATC.

The knockdown of KAT2A did not affect the XRCC5‐XRCC6 interaction. The K265R mutation abolished XRCC5 lactylation induced by KAT5 but not by KAT2A. These findings delineate a functional specificity within the lactylation landscape of XRCC5: while both KAT2A and KAT5 possess enzymatic activity toward XRCC5, only KAT5‐mediated lactylation at the K265 residue specifically stabilizes the XRCC6/XRCC5 heterodimer. Although KAT2A clearly modifies XRCC5, its effects appear to be independent of the K265 site and the subsequent stabilization of the XRCC6/XRCC5 heterodimer. KAT2A may thus regulate other aspects of XRCC5 biology. Therefore, the identification of KAT2A does not diminish the importance of KAT5‐mediated XRCC5 lactylation at K265; rather, it highlights that XRCC5 integrates multiple lactylation signals, among which KAT5‐mediated XRCC5 lactylation at K265 is the specific regulator of NHEJ hyperactivation through stabilizing the XRCC6/XRCC5 complex.​ Dissecting KAT2A‐mediated XRCC5 lactylation sites and their biological consequences warrants a dedicated future investigation.

Collectively, our findings and existing evidence demonstrate that Warburg effect‐mediated lactate accumulation acts as a key mechanism to hyperactivate NHEJ and HR, which likely contributes to the acquisition of chemoresistance in various cancers. In response to DNA damage, ATM‐dependent lactylation of MRE11 at K673, catalyzed by CBP, enhances DNA end resection and HR repair, thereby promoting chemoresistance in cancer cells [39]. TIP60‐mediated lactylation of NBS1 at K388, which facilitates MRE11–RAD50–NBS1 (MRN) complex assembly and HR protein recruitment to DSBs, is essential for promoting HR‐mediated DNA repair and conferring chemoresistance [40]. Lactylation of XLF at K288, catalyzed by GCN5, promotes its binding to Ku80, recruitment to DSB sites, and NHEJ activity, thereby conferring chemoresistance in cancer [22]. Consequently, these findings highlight hyperactive DNA repair pathways as potential investigational targets to address chemoresistance and improve treatment efficacy in several malignant tumors.

While oxamate has been reported to exert anti‐tumor effects in triple‐negative breast cancer (TNBC) [41], our data in ATC models (new Figures 1H and 6A) reveal limited monotherapy efficacy. This observation aligns with recent findings from Jiang et al. [39], where LDHA inhibition by oxamate similarly failed to suppress colorectal cancer (CRC) growth as a single agent; instead, it functioned primarily to sensitize tumors to DNA‐damaging agents. We attribute this discrepancy primarily to inherent biological differences between cancer types. The response to oxamate is highly context‐dependent, varying with specific genetic drivers and metabolic landscapes, and the specific factors determining this differential sensitivity remain an active area of investigation.

This study has several limitations. First, owing to the low incidence of ATC, samples from chemoresistant ATC patients are unavailable, which precludes confirmation of the relationship between XRCC5 lactylation at K265 and chemoresistance in ATC. Second, the ATC cohort used for IHC and immunofluorescence analyses was small (n = 7); although this reflects the natural incidence of this ultrarare malignancy at a single center, it limits definitive conclusions regarding prognostic stratification or treatment response correlations. Third, the generation of XRCC5 K265R mutant mice emerges as a critical step toward deconvoluting the in vivo mechanisms and physiological relevance of this modification. The absence of an XRCC5 K265R mouse model constrains our ability to assess the broader biological impact of XRCC5 K265 lactylation modification in vivo.

## 4 Materials and Methods

### 4.1 Clinical Samples

We recruited a cohort comprising 7 ATC and 10 benign thyroid nodule (BTN) patients, all initially diagnosed at the First Affiliated Hospital of Nanchang University between 2015 and 2024. Formalin‐fixed paraffin‐embedded (FFPE) specimens and corresponding clinicopathological data (including gender, age, histological subtype, and TNM stage) were obtained from the hospital's Pathology Department (Table S1). The tissue sections from all TC patients were independently reviewed by at least two experienced pathologists. All procedures were approved by the Medical Research Ethics Committee of the First Affiliated Hospital of Nanchang University as per the Helsinki Declaration.

### 4.2 Cell Culture

The HEK293T cells were cultured in Dulbecco's Modified Eagle's Medium (DMEM) supplemented with 10% fetal bovine serum (FBS; Gibco) and 1% penicillin‐streptomycin solution. The normal human thyroid cell line Nthy‐ori 3‐1 and human ATC cell lines CAL‐62 and 8305C were purchased from Procell (China). The PTC cell line TPC‐1 was obtained from Procell, while BCPAP was purchased from Fuheng Biology (China). All cell lines were authenticated via short tandem repeat (STR) profiling prior to experimentation. For routine culture, Nthy‐ori 3‐1 and TPC‐1 were maintained in RPMI 1640, BCPAP and CAL‐62 were cultured in DMEM, and 8305C was maintained in Minimum Essential Medium (MEM). All media were supplemented with 10% fetal bovine serum (FBS) and 1% penicillin‐streptomycin solution. The above cells were maintained at 37°C under 5% CO_2_ in a humidified atmosphere.

### 4.3 siRNA or Plasmid Transfection

The siRNA targeted for ANXA2 was obtained from Sangon Biotech (China) and transfected using RNATransMate (Sangon Biotech, China) according to the manufacturer's instructions. Plasmids overexpressing Flag‐tagged XRCC5 (wild‐type), Flag‐tagged LDHA, and Myc‐tagged ANXA2 were purchased from Miaoling Biology (China). The K265R mutant plasmid of XRCC5 was constructed by using QuickMutation Site‐Directed Mutagenesis Kit (Beyotime, China). Plasmids overexpressing His‐tagged KAT2A, KAT2B, KAT5, or KAT8 were obtained from WZ Biosciences (China). All plasmids were transfected using linear polyethylenimine (PEI; molecular weight 40,000) from Yeasen (China) according to the manufacturer's instructions.

### 4.4 Construction of Stable Cell Lines

To generate stable cell lines with XRCC5 knockdown or ANXA2 overexpression, lentiviral particles were first produced by co‐transfecting HEK293T cells with an XRCC5 knockdown or an ANXA2 overexpression construct and the packaging plasmids pMD2G and pSPAX2. The viral supernatant was harvested at 24 and 48 h post‐transfection, and was then used to transduce target cells in the presence of 8 µg/mL polybrene to enhance infection efficiency. Then, transfected cells were selected with puromycin. The sequences for the shRNAs against XRCC5 (shXRCC5), KAT2A (shKAT2A), KAT5 (shKAT5), and ANXA2 (shANXA2), are provided in Table S2. To obtain stable XRCC5 WT and K265R mutant cells, 8305C and CAL‐62 cells with stable XRCC5 knockdown were further infected with lentiviral‐XRCC5 WT and lentiviral‐XRCC5 K265R and then screened by G418.

### 4.5 Cell Counting Kit‐8 Assay

Sensitivity to chemotherapy was evaluated by quantifying cell viability using the Cell Counting Kit‐8 (CCK‐8, Abmole, USA). Cells were plated in a 96‐well plate at 2,000 cells per well. After 24 h, they were treated with the indicated compounds. Following a 72‐h treatment period, 10 µL of CCK‐8 reagent was added to each well, followed by a 1‐h incubation prior to absorbance measurement. The absorbance (optical density, OD) at 450 nm was subsequently measured using a Multiskan FC microplate reader (Thermo Scientific, USA).

### 4.6 Western Blotting

Cellular proteins were extracted using the Cell lysis buffer for Western and IP (Beyotime, China) containing 1 mM PMSF. After separation by SDS‐PAGE and transfer to a PVDF membrane, proteins were probed with primary antibodies, followed by an HRP‐conjugated secondary antibody. Signal detection was performed with the BeyoECL Star kit (Beyotime, China). The antibodies used for western blotting were listed in Table S3.

### 4.7 Co‐Immunoprecipitation

Protein extracts were prepared in Cell Lysis Buffer for Western and IP (Beyotime, China) supplemented with 1 mM PMSF. The cell supernatant was incubated overnight at 4°C with mouse anti‐Flag (66008‐4‐Ig, 5 µg, Proteintech), anti‐Myc (60003‐2‐Ig, 5 µg, Proteintech) antibody, followed by the addition of protein A/G beads (Beaver, China). After incubation, the beads were washed with washing buffer and then boiled in SDS‐PAGE loading buffer for subsequent western blotting analysis.

### 4.8 Mass Spectrometry

Cell lysates were prepared as described above and subjected to immunoprecipitation using a rabbit anti‐LDHA antibody (19987‐1‐AP; Proteintech) or IgG control. The antibody‐antigen complexes were captured with protein A/G beads (Beaver, China) and washed thoroughly. Bound proteins were eluted by boiling in SDS‐PAGE loading buffer, separated by SDS‐PAGE, and visualized using a Fast Silver Stain Kit (Beyotime, China) according to the manufacturer's protocol. Protein bands of interest were excised and analyzed by LC‐MS/MS (Sangon Biotech, China).

### 4.9 Immunohistochemistry (IHC)

IHC staining was conducted with the following primary antibodies: anti‐pan‐Klac, anti‐LDHA, anti‐P‐LDHA, anti‐ANXA2, and anti‐Ki67 (Table S3). Following staining, the slides were imaged using a microscope (Carl Zeiss, Oberkochen, Germany) and analyzed with ImageJ software (National Institutes of Health, Bethesda, MD, USA) and Image‐Pro Plus software (Media Cybernetics, Rockville, MD, USA).

### 4.10 Immunofluorescence Staining

FFPE sections from ATC or BTN patients underwent heat‐induced antigen retrieval at 65°C for 2 h, followed by deparaffinization in xylene and rehydration through a graded ethanol series (100%, 95%, 85%, 75%) ending with a distilled water rinse. Antigen retrieval was performed by boiling the sections in EDTA buffer (pH 9.0), after which they were washed three times with PBS. Endogenous peroxidase activity was blocked by incubation with 3% H_2_O_2_ for 10 min at room temperature. After three additional PBS washes, sections were incubated with 10% goat serum in PBS for 30 min at room temperature to block nonspecific binding. Primary antibodies against pan‐Klac and XRCC5 (Table S3) were diluted in blocking buffer and applied to the sections overnight at 4°C. Following primary antibody incubation, sections were washed and incubated with secondary antibodies conjugated to FITC‐495 and Alexa Fluor 594. Nuclei were counterstained with DAPI. All images were acquired using a high‐resolution laser scanning confocal microscope (Leica Stellaris 5).

### 4.11 Isolation of sEVs From Supernatant of Cell Culture

Prior to sEV isolation, cells were cultured in DMEM medium containing 10% sEV‐depleted FBS (EXO‐FBS; System Biosciences) for 3–4 days until reaching 90% confluency. The sEVs were isolated from culture media by differential ultracentrifugation. Briefly, the media was sequentially centrifuged at 3,000 × g for 10 min and 10 000 × g for 10 min (at 4°C) to remove cells and debris. The resulting supernatant was filtered through a 0.45 µm filter and then ultracentrifuged at 100 000 × g for 2 h at 4°C using an Optima XPN‐100 ultracentrifuge equipped with a 32Ti rotor (Beckman Coulter, Germany). The pellet was washed with cold, filtered PBS, followed by a second ultracentrifugation under the same conditions. The final sEV pellet was resuspended in 50–70 µL of PBS containing a protease inhibitor and stored at ‐80°C until use. The protein concentration of the isolated sEVs was quantified using a Pierce BCA assay kit (Thermo Scientific, USA). The presence of tumor susceptibility gene 101 (TSG101, #R25999, ZEN BIO, China) and Golgi Matrix 130 (GM130, #HA721282, HuaBio, China) in the isolated sEVs was analyzed by western blotting. The microphotographs of sEVs were obtained using an HT7700 transmission electron microscope (Hitachi, Japan).

### 4.12 Cell Uptake Experiment

Following labeling with 3,3′‐dioctadecyloxacarbocyanine perchlorate (DiO, 1:100 dilution; Beyotime, China), sEVs were washed twice with RPMI‐1640 medium, resuspended in PBS, and co‐cultured with 8305C or CAL‐62 cells for 24 h at 37°C. Post‐fixation with 4% paraformaldehyde, cells were stained with 4',6‐diamidino‐2‐phenylindole (DAPI, Beyotime, China). After a final PBS wash, sEV uptake was examined and imaged on a Carl Zeiss (Germany) inverted fluorescence microscope.

### 4.13 Proximity‐Dependent Barcoding Assay (PBA) and sEV Proteome Data Analysis

A library of PBA probes was utilized, each consisting of an antibody conjugated to a unique oligonucleotide barcode. For sEV capture, a 96‐well streptavidin‐coated plate was prepared by incubating each well with biotinylated cholera toxin subunit B (biotin‐CTB, 2.5 µg/mL; Thermo Scientific) for 20 min at room temperature, followed by three washes with PBST. The sEVs isolated from cell culture supernatant were incubated with the PBA probe library for 2 h. Subsequently, 20 µL of this mixture was transferred to the CTB‐coated plate. The sEVs were affinity‐captured via the specific binding of CTB to GM1 gangliosides enriched in the sEV membrane. Rolling circle amplification (RCA) products, each representing a single‐sEV barcode, were added to the plate and annealed to the oligonucleotides on the bound PBA probes. These oligonucleotides were then extended using the RCA products as templates. The extended products were harvested and used to construct sequencing libraries. Libraries were sequenced (PE150) on an Illumina NovaSeq S4 platform. Raw sequencing data (bcl files) were converted to fastq format using bcl2fastq (v2.20.0.42). Data analysis was performed using the EVisualizer decoding package (v1.0, Secretech). Briefly, raw reads were processed to identify sEVs and their protein cargo. The total expression level of each protein was summarized and integrated into a dataset. This dataset was normalized using the trimmed mean of M‐values (TMM) method to generate the standardized protein expression.

### 4.14 Measurement of Lactate Content

The lactate content in the cell was measured according to the manufacturer's instructions of the Lactic Acid Assay Kit (Solarbio, China). Absorbance at 570 nm was read using a spectrophotometer, and the lactate concentration was normalized to the cell number.

### 4.15 LDHA Enzyme Activity

Cells were lysed, and the total protein concentration was quantified via a BCA assay. For the enzymatic reaction, 5 mg of protein was combined with a solution consisting of 0.2 m Tris‐HCl (pH 7.3), 0.05% bovine serum albumin, 2 mM pyruvate, 10 mM MgCl_2_, and 20 mM NADH. The reaction was monitored by measuring the decrease in absorbance at 340 nm, which corresponds to the oxidation of NADH to NAD^+^, using a spectrophotometer.

### 4.16 Molecular Docking

The X‐ray crystal structures of XRCC5‐K265(1JEQ) and XRCC6(1JEQ) were retrieved from the Protein Data Bank. To ensure the accuracy of the docking results, the protein was prepared by the AutoDockTools‐1.5.7, and the water molecules were manually eliminated from the protein and the polar hydrogen was added. Docking Web Server (GRAMM) was used for protein‐protein docking. The resulting protein‐protein complex was also manually optimized by removing water and adding polar hydrogen by the AutoDockTools‐1.5.7. Finally, the protein‐protein interactions were predicted and the protein‐protein interaction figure was generated by PyMOL. The XRCC5 protein is represented as a slate cartoon model, XRCC6 protein is shown as a cyan cartoon model, and their binding sites are shown as the corresponding‐colored stick structure. When focusing on the binding region, the binding site is then shown as a presentation of the protein to which it belongs.

### 4.17 Extraction of Chromatin Fractions and Soluble Fractions

Cells were lysed on ice for 10 min in NETN buffer (20 mM Tris–HCl pH 8.0, 100 mM NaCl, 1 mM EDTA, and 0.5% Nonidet P‐40) supplemented with 1× protease inhibitors. Following centrifugation at 12 000 × g for 10 min at 4°C, the supernatant was collected as soluble fractions. The insoluble pellet was washed three times with PBS and then subjected to lysis in ice‐cold EBC2 buffer (50 mM Tris‐HCl pH 7.5, 300 mM NaCl, 5 mM CaCl_2_, 10 U micrococcal nuclease). After sonication and a second centrifugation (12 000 × g, 10 min, 4°C), the resulting supernatant was retained as the chromatin fraction.

### 4.18 Comet Assay

Cells were plated in 6‐well plates at a density of 1.0 × 10^5^ cells per well. Following cisplatin treatment as specified, cells were collected at designated time points and processed for the comet assay. In brief, harvested cells were suspended in low‐melting‐point agarose and applied to glass slides, followed by lysis in lysis buffer at 4°C for 1 h. The slides were subsequently incubated in 1× electrophoresis buffer for approximately 40 min to allow DNA unwinding. Electrophoresis was then conducted at 25 V for 25 min. After electrophoresis, samples were stained with propidium iodide and mounted with a coverslip. For analysis, comets were visualized under a fluorescence microscope, and DNA damage was assessed by measuring tail moment.

### 4.19 The Cellular Nhej Assays

To assess cellular NHEJ repair activity, 8305C and CAL‐62 cells were co‐transfected with the I‑SceI‑HA and EJ5‑GFP reporter plasmids using Lipofectamine 3000 (Thermo Scientific). After 48 h, we analyzed the percentage of GFP‑positive cells by flow cytometry on a BriCyte E6 system (MindRay, China).

### 4.20 Nuclear Protein Extraction

Twenty million cells (2.0 × 10^7^) of either 8305C or CAL‐62 cell lines were detached using trypsin, pelleted via centrifugation (2000 × g, 5 min), and washed twice with PBS. The resulting cell pellet was gently resuspended in five times its volume of ice‐cold hypotonic lysis buffer (10 mM HEPES, pH 7.5 at 4°C, 5 mM KCl, 1.5 mM MgCl_2_, 0.2 mM PMSF, 0.5 mM DTT) and incubated on ice for 10 min, followed by centrifugation at 1200 × g for 10 min. After discarding the supernatant, the pellet was resuspended in a volume of fresh hypotonic buffer equal to the pellet volume. Cells were lysed by mechanical disruption using a Dounce homogenizer with pestle B for 20 strokes. Following homogenization, 3 M KCl was added dropwise to the lysate to reach a final concentration of 50 mM KCl. The mixture was incubated on ice for 10 min and then centrifuged at 3000 × g for 20 min to pellet nuclei. The nuclear pellet was carefully resuspended in two volumes of low‐salt buffer (20 mM HEPES, pH 7.9 at 4°C, 1.5 mM MgCl_2_, 20 mM KCl, 0.2 mM EDTA, 0.2 mM PMSF, 0.5 mM DTT), followed by the addition of one volume of high‐salt buffer (10 mM HEPES, pH 7.5 at 4°C, 1.6 M KCl, 1.5 mM MgCl_2_). Following extraction in the salt buffer mixture with gentle agitation (4°C, 30 min), the nuclear lysate was centrifuged at 10 000 × g for 30 min at 4°C to pellet insoluble debris. The supernatant containing soluble nuclear proteins was retained for subsequent analysis.

### 4.21 The Cell‐Free Nhej Assays

Plasmid‐based DNA end‐joining assays were conducted to assess cell‐free NHEJ repair activity as previously described [42]. In each 20 µL reaction mixture containing 20 mM HEPES (pH 7.5), 80 mM KCl, 10 mM MgCl_2_, 1 mM ATP, and 1 mM DTT, 10 µg of the extracted nuclear protein was incubated with 200 ng of BamHI‐linearized pSP65 plasmid (3005 bp) for 1 h at 25°C. Reactions were terminated by adding a stop solution containing 1 µL of 10 mg/mL proteinase K, 2 µL of 0.5 M EDTA (pH 7.5), and 2 µl of 0.5% SDS, followed by incubation for 30 min at 37°C. Finally, DNA products were resolved by agarose gel electrophoresis.

### 4.22 Animal Experiments

All animal procedures were performed following approval from the Animal Ethics Committee of the First Affiliated Hospital of Nanchang University (No. CDYFY‑IACUC‑202510GR008). Six‑week‑old male BALB/c nude mice, purchased from Hangzhou Ziyuan Laboratory Animal Technology Co., Ltd. (China), were maintained in a specific pathogen‑free environment throughout the study. A suspension of 1.0 × 10^6^ 8305C cells was prepared in a 1:1 mixture of serum‐free RPMI 1640 medium and Matrigel (BD Biosciences), with a total volume of 100 µL. This suspension was administered to the mice via subcutaneous injection. Twenty‐four hours post‐inoculation, the mice were randomly divided into various experimental groups: (1) vehicle (saline); (2) oxamate (750 mg/kg, daily); (3) cisplatin (2 mg/kg, once a week); (4) a combination of the two agents at the doses specified above.

To explore the function of XRCC5 lactylation at K265 in ATC chemoresistance, we established a xenograft model by subcutaneously injecting nude mice with 1.0 × 10^6^ 8305C cells. These cells featured endogenous XRCC5 knockdown and stable expression of either XRCC5 WT or the K265R mutant. The mice were randomly allocated to the following groups: (1) vehicle (saline) + XRCC5 WT; (2) vehicle + XRCC5 K265R; (3) cisplatin + XRCC5 WT; (4) cisplatin + XRCC5 K265R.

Tumor volume was monitored by caliper measurements throughout the study. On day 27, all mice were euthanized, and tumors were harvested. The final tumor volume was measured, and the tumors were weighed.

### 4.23 Statistical Analysis

Statistical analysis was conducted with GraphPad Prism 8.0 software. Data are presented as mean ± SD from three independent experiments, unless otherwise noted. For comparisons between two groups, significance was assessed using the student's t‐test. Comparisons involving multiple groups were evaluated by one‐way or two‐way ANOVA analysis. A *p* value of less than 0.05 was considered statistically significant.

## Author Contributions

S.S.S. and X.Y.S. contributed equally to this study. D.D.F. and M.X. designed the study. S.S.S., X.Y.S., and L.Y.X. conducted the experiments. S.S.S. and D.D.F. analyzed the data. D.D.F. and M.X. supervised the study. D.D.F. provided funding support. D.D.F. wrote the manuscript. All authors have reviewed and approved the paper.

## Ethics Approval and Consent to Participate

This study was approved by the Medical Research Ethics Committee of the First Affiliated Hospital of Nanchang University (2026)CDYFYYLK(01‐024). The requirement for informed consent for this study was waived due to the retrospective nature of the study and the use of archival FFPE tissues stored in the Department of Pathology. All procedures were conducted in accordance with the ethical standards of the Helsinki Declaration. All animal experimental protocols were approved by the Animal Ethics Committee of the First Affiliated Hospital of Nanchang University (No. CDYFY‑IACUC‑202510GR008).

## Consent for Publication

All co‐authors have given their consent to the submitted version of the manuscript for publication.

## Conflicts of Interest

The authors declare no conflicts of interest.

## Supporting information




**Supporting File 1**: advs76402‐sup‐0001‐SuppMat.docx.

**Supporting File 2**: advs76402‐sup‐0002‐TableS1.docx.

**Supporting File 3**: advs76402‐sup‐0003‐TableS2.docx.

**Supporting File 4**: advs76402‐sup‐0004‐TableS3.docx.

## Data Availability

The data that support the findings of this study are available from the corresponding author upon reasonable request.
