# Supplementary material for: ANXA2+ Small Extracellular Vesicles Drive Chemoresistance in Anaplastic Thyroid Cancer by Promoting XRCC5 Lactylation and Enhancing Non‐Homologous End‐Joining Repair
Source: Adv Sci (Weinh). 2026 Jul 3;13(54):e76402. doi: 10.1002/advs.76402 (PMC13334595; doi:10.1002/advs.76402)
Supplement: Supplementary file 2 [file ADVS-13-e76402-s002.docx]

**Table S2. The sequence of the shRNAs used in this study.**

| **Name** | **Sequence** |
| --- | --- |
| shXRCC5 | AGAGGAAGCCTCTGGAAGTTCTTCAAGAGAGAACTTCCAGAGGCTTCCTCT |
| shKAT2A | CGATGTTCGAGCTCTCAAAGATTCAAGAGATCTTTGAGAGCTCGAACATCG |
| shKAT5 | TCGAATTGTTTGGGCACTGATTCAAGAGATCAGTGCCCAAACAATTCGA |
| shANXA2 | GAGTCTACAAGGAAATGTACATTCAAGAGATGTACATTTCCTTGTAGACTC |
