# Supplementary material for: ANXA2+ Small Extracellular Vesicles Drive Chemoresistance in Anaplastic Thyroid Cancer by Promoting XRCC5 Lactylation and Enhancing Non‐Homologous End‐Joining Repair
Source: Adv Sci (Weinh). 2026 Jul 3;13(54):e76402. doi: 10.1002/advs.76402 (PMC13334595; doi:10.1002/advs.76402)
Supplement: Supplementary file 3 [file ADVS-13-e76402-s003.docx]

**Table S3. Antibodies use for Western blotting and Immunohistochemistry in this study.**

| **WB** | | | |
| --- | --- | --- | --- |
| **Antibodies** | **Source** | **Identifier** | **Dilution** |
| Anti-L-Lactyl lysine | PTM Bio | PTM-1401RM | 1:1000 |
| Anti-beta-actin | Proteintech | 66009-1-Ig | 1:1000 |
| Anti-Flag | Proteintech | 20543-1-AP | 1:2000 |
| Anti-XRCC5 | Upingbio | YP-mAb-00426 | 1:1000 |
| Anti-XRCC6 | ZENBIO | R26942 | 1:1000 |
| Anti-Myc | Proteintech | 16286-1-AP | 1:4000 |
| Anti-H3 | Proteintech | 17168-1-AP | 1:1000 |
| Anti-His | HUABIO | HA722798 | 1:2000 |
| Anti-Src | ZENBIO | R 25792 | 1:1000 |
| Anti-LDHA | Proteintech | 19987-1-AP | 1:5000 |
| Anti-P-SRC | ABclonal | AP1027 | 1:1000 |
| Anti-P-LDHA | Immunoway | YP1385 | 1:1000 |
| Goat Anti-Mouse IgG(H+L) | Proteintech | SA00001-1 | 1:10000 |
| Goat Anti-Rabbit IgG(H+L) | Proteintech | SA00001-2 | 1:10000 |
| Anti-TSG101 | ZENBIO | R25999 | 1:2000 |
| Anti-GM130 | HUABIO | HA721282 | 1:1000 |
| Anti-ANXA2 | Proteintech | 11256-1-AP | 1:1000 |
| **Immunohistochemistry** | | | |
| **Antibodies** | **Source** | **Identifier** | **Dilution** |
| Anti-LDHA | Proteintech | 19987-1-AP | 1:200 |
| Anti-P-LDHA | Immunoway | YP1385 | 1:100 |
| Anti-L-Lactyl lysine | PTM Bio | PTM-1401RM | 1:100 |
| Anti-ANXA2 | Proteintech | 11256-1-AP | 1:500 |
| Anti-Ki67 | HUABIO | HA721115 | 1:5000 |
| **Immunofluorescence** | | | |
| **Antibodies** | **Source** | **Identifier** | **Dilution** |
| Anti-XRCC5 | Proteintech | 66546-1-Ig | 1:300 |
| Anti-L-Lactyl lysine | PTM Bio | PTM-1401RM | 1:200 |
