# Supplementary material for: ANXA2+ Small Extracellular Vesicles Drive Chemoresistance in Anaplastic Thyroid Cancer by Promoting XRCC5 Lactylation and Enhancing Non‐Homologous End‐Joining Repair
Source: Adv Sci (Weinh). 2026 Jul 3;13(54):e76402. doi: 10.1002/advs.76402 (PMC13334595; doi:10.1002/advs.76402)
Supplement: Supplementary file 4 [file ADVS-13-e76402-s004.docx]

**Table S1.** **The clinicopathological features of patients recruited in this study.**

| **ID** | **Gender** | **Age** | **Histological subtype** | **TNM stage** | **Clinical stage** | **Treatment  received** | **Follow-up (months)** | **Survival**  **status** |  |
| --- | --- | --- | --- | --- | --- | --- | --- | --- | --- |
| P01 | Female | 77 | ATC | T2N1bM0 | IVB | Thyroidectomy surgery | 2 | Dead |  |
| P02 | Female | 74 | ATC | T2NxM0 | IVA | Thyroidectomy surgery | 8 | Dead |  |
| P03 | Female | 71 | ATC | T4aN1M0 | IVB | Thyroidectomy surgery | 4 | Dead |  |
| P04 | Female | 59 | ATC | T4aN1bM0 | IVB | Thyroidectomy surgery | Lost to follow-up | Unknown |  |
| P05 | Male | 74 | ATC | T2N1aM0 | IVB | Thyroidectomy surgery | 5 | Dead |  |
| P06 | Female | 69 | ATC | T4aN0M0 | IVB | Thyroidectomy surgery | Lost to follow-up | Unknown |  |
| P07 | Male | 68 | ATC | T3bN0M0 | IVB | Thyroidectomy surgery | 6 | Dead |  |
| P8 | Male | 63 | BTN |  |  |  |  |  |  |
| P9 | Male | 63 | BTN |  |  |  |  |  |  |
| P10 | Female | 75 | BTN |  |  |  |  |  |  |
| P11 | Male | 47 | BTN |  |  |  |  |  |  |
| P12 | Female | 51 | BTN |  |  |  |  |  |  |
| P13 | Female | 69 | BTN |  |  |  |  |  |  |
| P14 | Female | 53 | BTN |  |  |  |  |  |  |
| P15 | Female | 69 | BTN |  |  |  |  |  |  |
| P16 | Female | 50 | BTN |  |  |  |  |  |  |
| P17 | Female | 37 | BTN |  |  |  |  |  |  |

ATC, anaplastic thyroid cancer; BTN, benign thyroid nodule.
