# Supplementary material for: ANXA2+ Small Extracellular Vesicles Drive Chemoresistance in Anaplastic Thyroid Cancer by Promoting XRCC5 Lactylation and Enhancing Non‐Homologous End‐Joining Repair
Source: Adv Sci (Weinh). 2026 Jul 3:e76402. Online ahead of print. doi: 10.1002/advs.76402 (PMC13334595; doi:10.1002/advs.76402)
Supplement: Supplementary file 1 — Supporting File 1: advs76402‐sup‐0001‐SuppMat.docx. [file ADVS-9999-e76402-s001.docx]

**ANXA2^+^ Small Extracellular Vesicles Drive Chemoresistance in Anaplastic Thyroid Cancer by Promoting XRCC5 Lactylation and Enhancing non-homologous end-joining Repair**

Shanshan Su, Yi Shan Xiong, Yuxuan Liang, Xiang Min*****, and Daofeng Dai*****


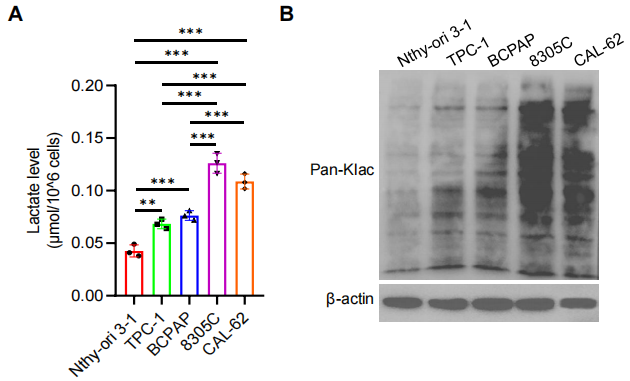


**Figure S1. Comparison of lactate production and pan-lactylation levels in normal, PTC, and ATC cells.** Lactate production **(A)** and pan-lactylation **(B)** levels in normal thyroid epithelial cell (Nthy-ori 3-1), PTC cells (TPC-1 and BCPAP), and ATC cells (8305C and CAL-62). The data of lactate level was analyzed using one-way ANOVA (n = 3 per group). PTC, papillary thyroid cancer; ATC, anaplastic thyroid cancer.


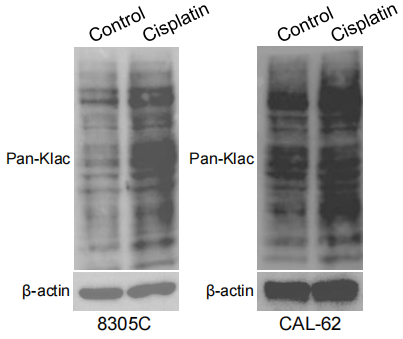


**Figure S2. The effect of cisplatin treatment on pan-lactylation levels in ATC cells.** Pan-lactylation levels in 8305C and CAL-62 cells treated with cisplatin (5 μM) for 24 h.


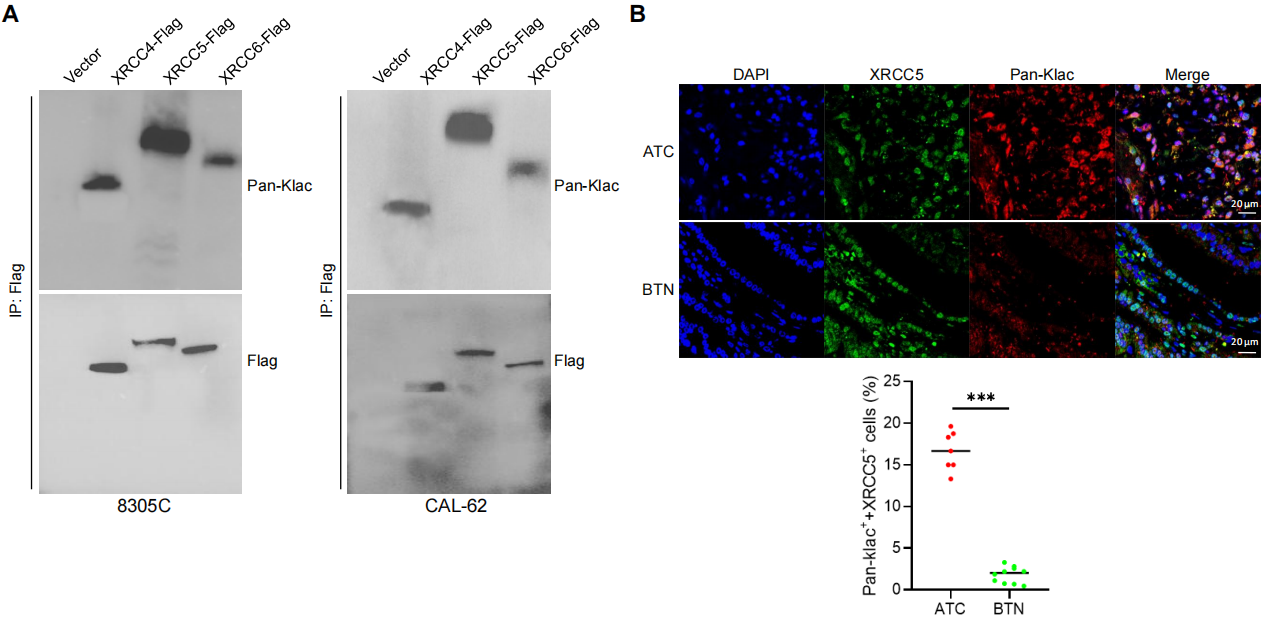


**Figure S3. Analysis of XRCC5 lactylation in ATC cells and tissues. (A)** Lysates from 8305C and CAL-62 cells overexpressing the indicated XRCC4-Flag, XRCC5-Flag, and XRCC6-Flag were immunoprecipitated with an anti-Flag antibody, followed by immunoblotting with the antibody against pan-Klac or the Flag tag. **(B)** Immunofluorescence assays showing co-localization of XRCC5 (Green) with pan-lactylation (Red) in ATC ( n = 7) and BTN ( n = 10) tissues. Five randomly selected fields were analyzed, and cells double‑positive for XRCC5 and pan‑lactylation were counted. DAPI‑positive cells were also quantified. The results are expressed as the ratio of XRCC5 and pan‑lactylation double‑positive cells to DAPI‑positive cells. BTN, benign thyroid nodule.

**
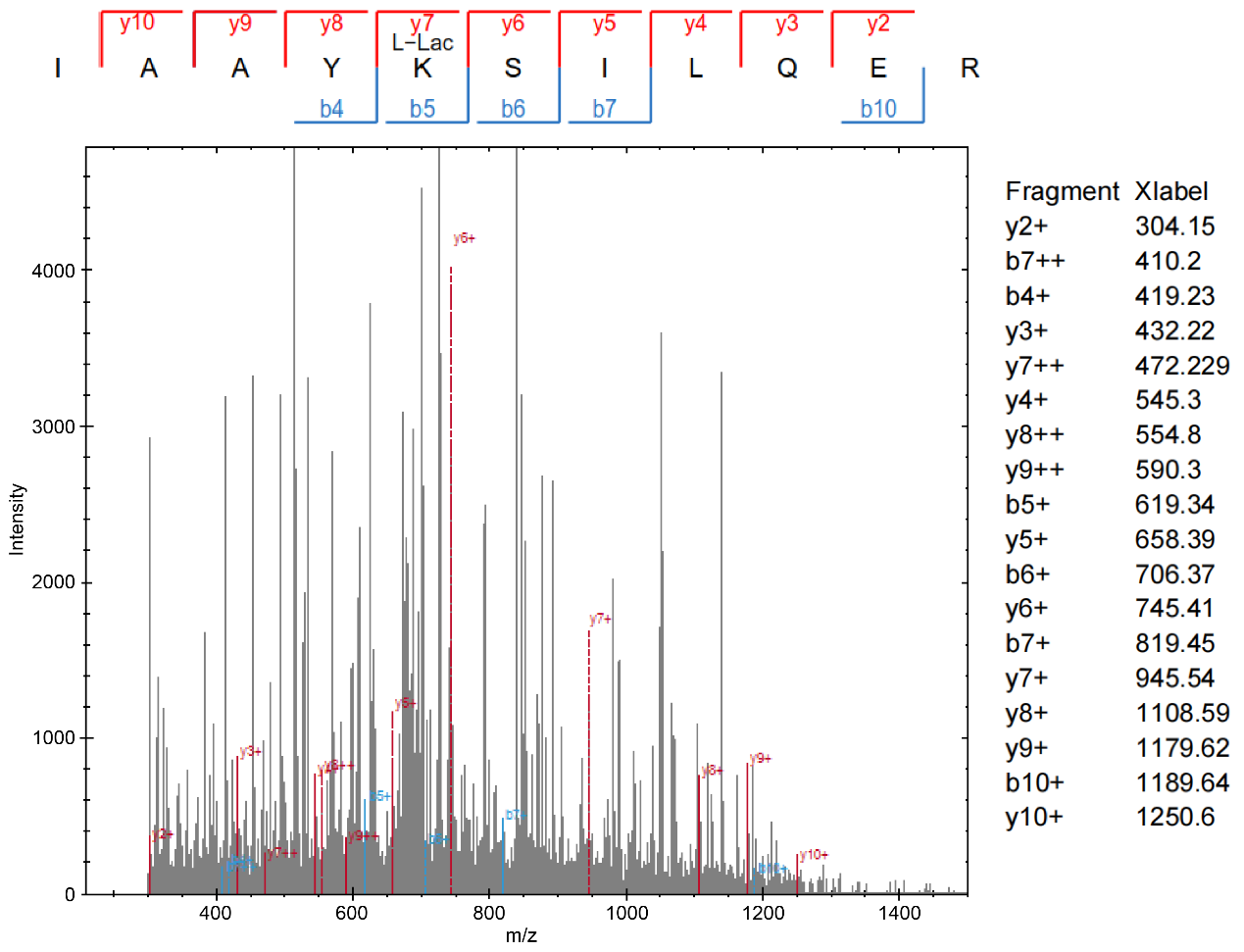
**

**Figure S4. The Mass spectra of XRCC5 lactylation at K265.** Using Spectronaut (v.18), we processed the acquired MS/MS spectra by searching against the Homo_sapiens_9606_SP_20231220.fasta (20,429 entries) concatenated with a reverse decoy database. Proteolytic cleavage was specified to be Trypsin/P, permitting up to 4 missed cleavages. The precursor mass error was set as 12.7 ppm. We specified carbamidomethyl on cysteine as a fixed modification, alongside variable modifications for protein N-terminal acetylation, methionine oxidation, and L-lactylation. The sequence coverage was 48%. Data were filtered to maintain a <1% false discovery rate.


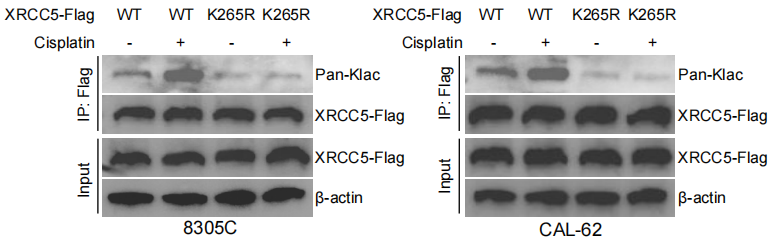


**Figure S5. The effect of cisplatin treatment on XRCC5 K265 lactylation in ATC cells.** Whole-cell extracts from 8305C and CAL-62 cells expressing XRCC5 WT or the XRCC5 K265R mutant, treated with control or cisplatin (5 μM) for 24 h, were immunoprecipitated with an anti-Flag antibody, followed by immunoblotting with the antibody against pan-Klac or the Flag tag.


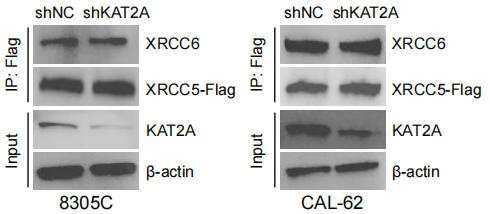


**Figure S6. The effect of depleting KAT2A on the interaction between XRCC5 and XRCC6 in ATC cells.** Cell lysates of 8305C and CAL-62 cells with or without stable knockdown of KAT2A were immunoprecipitated with an anti-Flag antibody, followed by immunoblotting with the antibody against XRCC6 or the Flag tag.


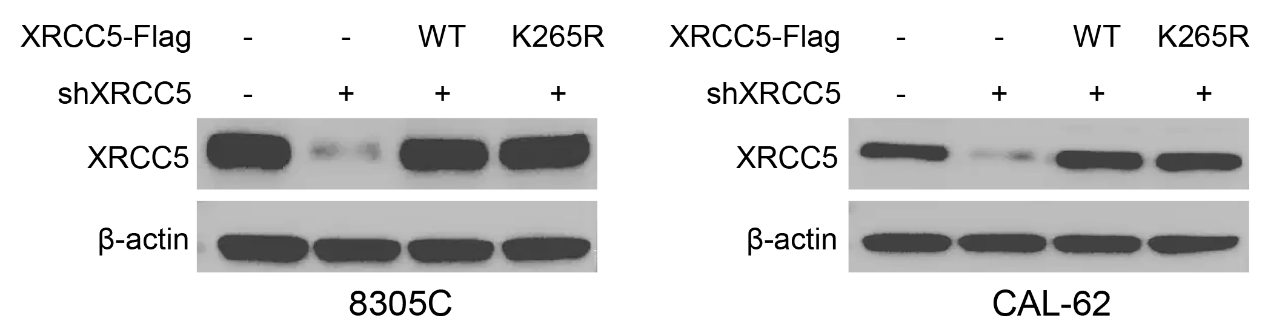


**Figure S7. Analysis of XRCC5 expression in ATC cells.** The expression of XRCC5 in ATC cells in which endogenous XRCC5 was knocked down and stably expressed either XRCC5 WT or the XRCC5 K265R mutant.


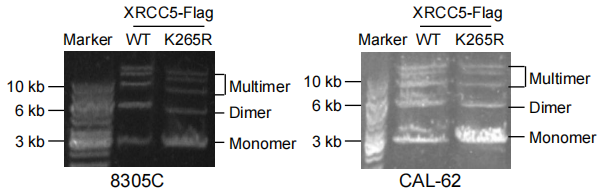


**Figure S8. The effect of XRCC5 K265R mutation on cell-free NHEJ activity in ATC cells.** The biochemical NHEJ assay was performed in 8305C and CAL-62 cells in which endogenous XRCC5 was knocked down and stably expressed either XRCC5 WT or the XRCC5 K265R mutant.


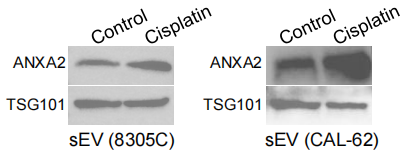


**Figure S9.** **The effect of cisplatin treatment on the secretion of ANXA2+ sEVs in ATC cells.** The sEVs were isolated from 8305C and CAL-62 cells treated with cisplatin (5 μM) for 24 h. The lysates of the isolated sEVs​ were then subjected to immunoblotting with antibodies against ANXA2 or TSG101. TSG101 was used as a loading control.


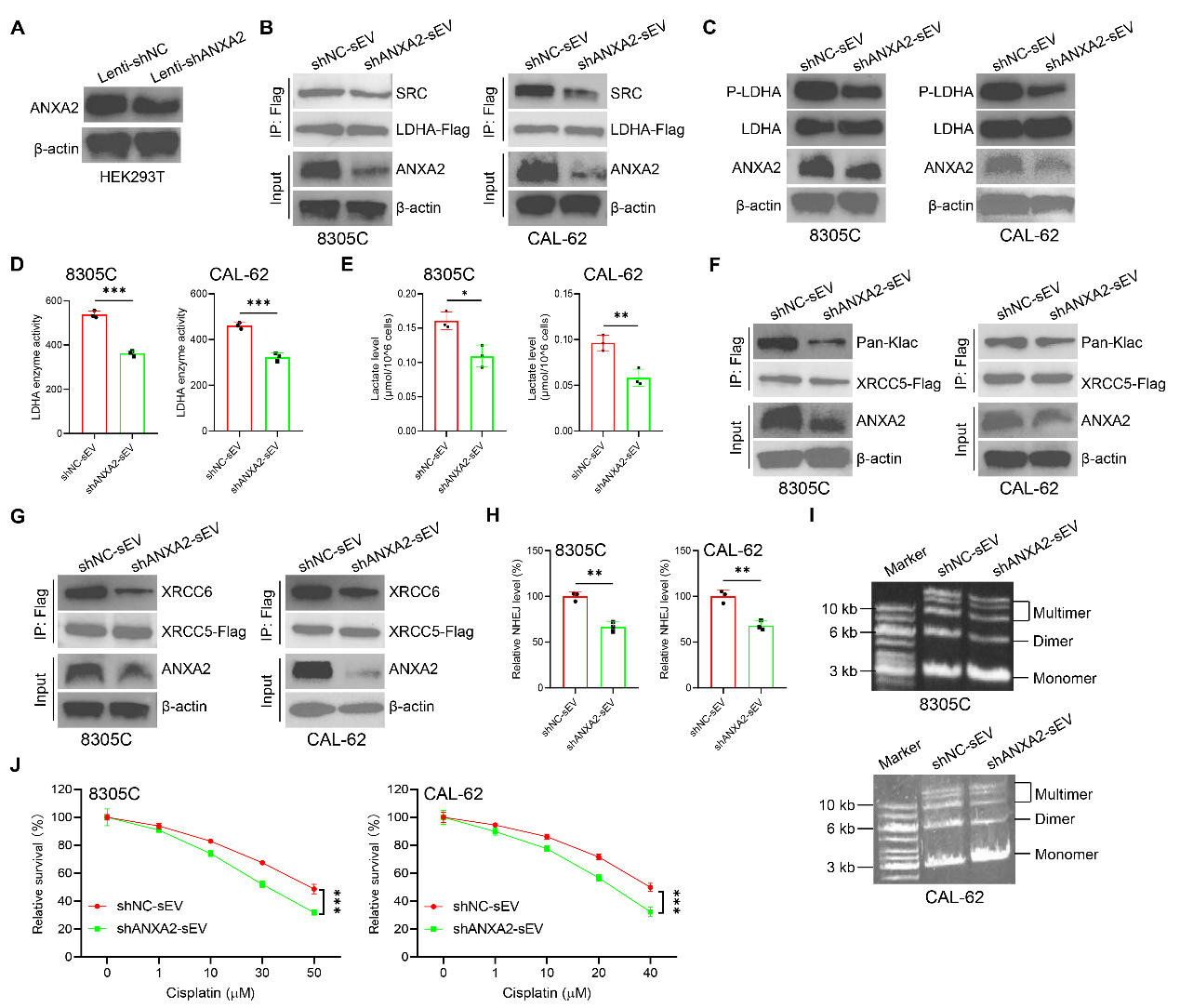


**Figure S10. The shANXA2-sEV reduces NHEJ repair and chemoresistance by decreasing the stability of the SRC/LDHA complex.** **(A)** The expression of ANXA2 in HEK293T cells stably transduced with lentiviral shANXA2 (Lenti-shANAX2) or lentiviral shNC (Lenti-shNC). shANXA2, the shRNA targeting ANXA2. NC, negative control. The 8305C and CAL-62 cells were treated with shNC-sEV or shANXA2-sEV. The effect of the above treatment on the interaction between SRC and LDHA **(B)**, LDHA phosphorylation at Y10 **(C)**, LDHA enzyme activity **(D)**, lactate production **(E)**, lactylation of XRCC5 **(F),** the interaction of XRCC5 with XRCC6 **(G)**, the cellular NHEJ repair efficiency **(H)**, the cell-free NHEJ activity **(I)**, and cisplatin resistance **(J)** in ATC cells. For panels D, E, and H, n = 3 per group; the student’s t-test was used.​ For panel J, n = 3 per condition; two-way ANOVA was used.
